# A health policy for hearing impairment in older Australians: what should it include?

**DOI:** 10.1186/1743-8462-2-31

**Published:** 2005-12-13

**Authors:** Jennifer L Smith, Paul Mitchell, Jie Jin Wang, Stephen R Leeder

**Affiliations:** 1 Australian Health Policy Institute, The University of Sydney, Victor Coppleson Building D02, NSW 2006, Australia; 2 Centre for Vision Research, The University of Sydney, Westmead Hospital, Hawkesbury rd, Westmead, NSW 2193, Australia; 3 Centre for Vision Research, The University of Sydney, Westmead Hospital, Hawkesbury rd, Westmead, NSW 2193, Australia; 4 Australian Health Policy Institute, The University of Sydney, Victor Coppleson Building D02, NSW 2006, Australia

## Abstract

**Background:**

As in all western countries, Australia's older population experiences high levels of hearing impairment coupled with relatively low levels of hearing device usage. Poor hearing diminishes the quality of life of affected individuals and their families. This paper discusses how to improve Australian hearing health policies in order to better combat this impairment amongst older Australians.

**Method:**

We searched the databases *Medline*, *Meditext *and *Web of Science *to find articles that discussed strategies and innovations to assist the hearing health of older people, and related this material to observations made during the Blue Mountains Hearing Study in NSW between 1997 and 2003.

**Results and Discussion:**

The literature search identified five areas for inclusion in a comprehensive hearing health policy in Australia. These are: early intervention; addressing of hearing aid expense; the use of assisted listening devices; hearing rehabilitation, and; screening and education. Further research in Australia is critical if we are to develop a strong approach to the increasing prevalence of age-related hearing loss.

**Conclusion:**

Australia needs to act now to address hearing impairment as it is a major cause of disability in those aged 55 and over. Federal and State governments should collaborate to construct a comprehensive hearing health policy that tackles poor levels of hearing health through early intervention, addressing hearing aid expense, encouraging the use of assisted listening devices, rehabilitation, screening and education. A good start would be to declare age related hearing impairment as a National Health Priority Area.

## Introduction

Policy-making around hearing health in Australia needs not only commitment but creativity. High rates of hearing impairment among older people, coupled with low rates of hearing aid use, demonstrate that the current policy approach is not working. This is alarming in a society whose population is ageing, as hearing loss is strongly associated with age: in Australia it affects 50% of the population aged 55 and over [1]. There are currently about 1.5 million Australians aged over 55 with some degree of bilateral hearing loss [1,2]. Wilson et al. (1999) state that this figure ranks hearing impairment amongst the most common disabilities in Australia [2]. As a cause of burden of disease, hearing impairment is the second-highest ranked disability for men in Australia, representing an average 5.7 years of life with a disability (YLD) for every Australian man [3]. For women it ranks eighth for disability, at 2.6 YLD [3].

The Federal Government has to some extent recognised hearing loss as a growing problem for Australia as the population ages, and has increased funding to hearing services provision [4]. But policy responses across Australia are still inadequate in sum, uncoordinated, under-researched and miss vulnerable sectors of the population in need of treatment [2]. Currently, the Federal Government supports 400,000 people through its Australian Government Hearing Services Program but this is barely one third of those in need of such services [2,4] and the adequacy of the response and support (matching new technologies to client need) is in serious question. The Government-funded Australian Hearing supports a research program through the National Acoustic Laboratories, especially hearing assessment, rehabilitation devices and hearing loss prevention. It does not, however, conduct or sponsor hearing *policy *research. Given the Federal Government's backing of 'Ageing Well, Ageing Productively' as a research priority "to develop better social, medical and population health strategies to improve the mental and physical capacities of ageing people", more attention needs to be paid to the identification and treatment of hearing impairment in older people [5].

Australia needs a stronger commitment to better hearing among older people through an effective and cohesive, national policy on hearing health. This policy needs the backing of a strong research program to provide a firm empirical base – for both its development and its evaluation.

This paper will now examine areas that are important in the development of a well-rounded hearing health policy and identify strategies that might lead to the creation of a comprehensive, effective approach to age-related hearing loss in Australia.

## Methods

We conducted a literature search to identify what should be included in a hearing health policy for older people for the next ten years for Australia. We searched the databases *Medline*, *Meditext *and *Web of Science *for articles about age-related hearing loss, hearing policy and hearing aids published during the past ten years. 555 articles were identified. We perused the abstracts of these articles and obtained the full text of relevant articles. From there, we used a 'snowballing' technique and looked for relevant articles from the reference lists of the papers not captured in the database search. Using an online search, we identified Australian state and federal government policy documents and guidelines and policy papers from the major hearing lobby groups in Australia, such as the Deafness Forum and Better Hearing.

Five areas emerged as important for the construction of a hearing health policy for older Australians. These areas were: early intervention; addressing of hearing aid expense; the use of assisted listening devices; the construction of a wide-ranging rehabilitation program, and; screening and education.

We also sought to identify areas which, while addressed in literature from overseas, provided no specific research data with respect to the Australian population.

## Results and discussion

### The impact of hearing loss on quality of life

That hearing loss is associated with poor quality of life among older people has been firmly established [6-11]. Even mild hearing impairment negatively affects many aspects of an individual's life, including interpersonal relationships, communication, socialisation and independence, and can lead to poor general health and mood disorders such as depression and anxiety [7-10]. It also leads to a greater reliance on community support services and may contribute to early aged care placement [1,12].

By contrast, with hearing loss, the successful use of hearing aids has been shown to be associated with a significant improvement in both quality and length of life among the hearing impaired [13,7,15,16]. Kochkin and Rogin (2000) conducted research on the U.S. population. They surveyed 2069 hearing-impaired individuals and 1710 people who had a family member who was hearing-impaired [7]. Those whose hearing was impaired and who used a hearing aid reported improved mood, physical health and social activity as compared to non-users of such devices [7].

These findings are supported by other smaller studies, which have also demonstrated similar improvements [16,17]. In addition, the family members of older hearing impaired people also report improved quality of life after their family member is fitted with a hearing aid [18].

### Poor use of hearing aids both in Australia and overseas

Despite evidence for a link between increased life quality and hearing aid use by older people with a hearing impairment, both the initial uptake and successful use of hearing devices amongst this group remain low [19-22]. A study of 1629 individuals aged 48 to 95 in the U.S. found that only 20% of those with hearing loss ever used a hearing aid, and that 29% of those people who had a hearing aid no longer used it [22]. In a study of 454 people aged 85 years and older in the Netherlands, Gussekloo et al. (2003) found that only one in three of those with severe hearing loss used a hearing aid [19]. Likewise, a UK study found that of 3452 people over 75 who failed a whispered voice hearing test, more than half did not own a hearing aid and only 60% of those who did used it regularly [23].

The data from Australian studies into hearing also demonstrate patchy hearing aid use and are largely comparable to those found in studies overseas [1,9,12]. The Australian Blue Mountains Hearing Study, which assessed the hearing of 2956 people aged 49 and over in the Blue Mountains region west of Sydney between 1997 and 2003 found that only 50% of older persons with moderate or worse levels of measured hearing loss had hearing aids (Table 1). Large scale qualitative research, which considers barriers to hearing aid usage from the perspective of the Australian population is, however, lacking. This deficit needs to be tackled as a prelude to policy changes that accurately address Australian needs.

**Table 1:** Proportions (numbers) of participants with hearing aid fitting and habitual use of hearing aid by the level of measured hearing loss (decibel hearing level, dB HL) in the better ear in the Blue Mountains Hearing Study population (aged 49 years or older).

Hearing aid fitting and habitual use	***Level of hearing loss (dBHL) in the better ear***		
	
	***Mild 26 ≤ 40dBHL***	***Moderate 41 ≤ 60dBHL***	***Severe >60dBHL***
**Women with fittings**	15.0 (51/340)	55.9 (71/127)	100.0 (19/19)
**Habitual use**	10.3 (35/340)	43.0 (55/128)	95.0 (19/20)
**Men with fittings**	18.1 (57/315)	54.1 (72/133)	87.1 (27/31)
**Habitual use**	14.6 (46/316)	43.3 (58/134)	64.5 (20/31)
**Persons with fitting**	16.5 (108/655)	55.0 (143/260)	92.0 (46/50)
**Habitual use**	12.4 (81/656)	43.1 (113/262)	76.5 (39/51)

We will now discuss factors that have been identified in the literature on age-related hearing loss as critical concerns for the development of a comprehensive policy approach to this problem.

### Early intervention

Earlier intervention for hearing loss through the provision of hearing rehabilitation and/or hearing aids would benefit older people and their families [24], especially if the hearing aids were of state-of-the-art digital design. Australian Hearing conducted research in 1999 that found that older hearing impaired individuals wait six to ten years before they seek help [15], as in the U.S. [11]. This is bad news for several reasons.

First, auditory deprivation, an inability to adequately recognize speech, may develop as a result of reduced auditory stimulation of the central auditory system [15,18,25]. It is only partially recoverable once hearing aids have been fitted [15]. Early intervention has also been said to contribute to the arrest of cognitive decline and communication problems [18].

Second, delayed hearing rehabilitation contributes to the lack of hearing aid retention and use in older people. If a hearing impaired person starts using a hearing aid at an earlier stage, they gain greater benefit from the device [15,26]. This is thought to be because the longer potential aid users wait, the greater the chance there is of communication difficulties, but more research is needed to confirm the significance of this factor [26]. Earlier use of hearing aids may also minimize damage to intimate and social relationships for hearing impaired individuals [7,10,11,28]. Age-related visual and dexterity problems also interfere with an individual's ability to learn to manage a hearing aid (twiddling the little knobs and switches), and a younger user learns these skills more easily and effectively [27]. Early intervention is therefore critical not just for the hearing impaired individual, but also for those around them [18].

Reasons why older individuals may not seek hearing aids at an earlier age include expense, a lack of education about the benefits of both hearing aids and assisted hearing devices and an underestimation (including denial) of the level of their hearing impairment [29]. Limited screening and case-finding programs, with inadequate referral and follow-up, also contribute to limited uptake and retention of hearing aids. These factors will be discussed in further detail below.

### Hearing aid expense

Prohibitive costs are associated with the poor use of hearing devices amongst the elderly Australian population [29], especially those of the superior digital selective compensatory devices. In Australia, Medicare does not cover the cost of hearing aids and free hearing aids are only provided for those over 60 who hold a Pensioner Concession Card [30]. The minimum cost for a basic, non-selective hearing aid and associated aural rehabilitation is around $1200 [31,32]. For common binaural deafness, the cost is double [31]. Selective compensatory digital devices cost around $6000 a pair.

Ching et al. (2003) suggested that the cost is hard to justify for many older people, especially pensioners [29]. The cost of hearing aids may mean that some people purchase only one aid, which causes problems because monaural fitting for binaural hearing loss is less effective, is associated with greater risk of auditory deprivation and may lead to a decrease in the variety of sound levels perceived. [18,25,33,34]. Surprisingly, however, overseas research reveals little difference in hearing aid uptake among countries that publicly fund hearing aids and those that do not, such as the U.S., though further research is needed in Australia [35]. Problems related to hearing aid expense will grow as the number of self-funded retirees increases in Australia.

### Assisted listening devices

When hearing aids alone cannot address hearing impairment, assisted listening devices can be used either on their own or in conjunction with hearing aids [15,28,36,38]. Assisted listening devices include the provision of extra loud, vibrating or visual-alerting devices, microphones, personal wireless systems and other devices [16,25,38].

Several authors have stated that proposing hearing aids as the preferred solution for hearing impairment amongst the elderly population is inadequate and ignores the value of other strategies [9,36,37]. The current range of hearing aids is not practical for a significant sector of the elderly population, such as those with poor manual dexterity because of arthritis or reduced cognitive functioning [16,36]. However, in Australia, as Hogan et al. (2001) discuss, apart from hearing aids, there are few alternative hearing devices and services routinely available to older people from the wide range of devices in existence [9].

For people who have problems with visual or manual dexterity or who need hearing assistance in certain situations in which hearing aids function poorly (such as nursing homes), assisted listening devices can be more appropriate [16,36,38]. Currently, most Commonwealth Hearing Services clients must pay for such devices or have them specially approved [28,39,40].

Government policies need to enable the more widespread use of assisted listening devices and offer rehabilitation services and greater subsidies for these devices [9,16].

### Counselling and rehabilitation programs

Hearing impairment requires service as well as technology. Both Australian and international studies demonstrate the benefit of counselling and hearing rehabilitation programs to supplement the provision of hearing aids [2,9,11,22,29,34,38,41,42,27]. Citron (2000) goes so far as to state that counselling is "the most overlooked aspect of the process of fitting amplification" and the "most important professional service an audiologist can provide for patients and their families" [44]. Welsh and Purdy (2001) state that hearing aids may be the first, but not the final, step in meeting the hearing needs of older people, and that hearing rehabilitation programs should be multi-faceted and explore alternatives to hearing aids [15].

Hearing rehabilitation programs should operate both pre- and post-hearing aid fitting and address other non-hearing aid focused approaches [36,42]. They should include training for hearing aid manipulation, which assists those who have problems with manual dexterity and vision [27,45]. Lesner (2003:s74) writes that the use of assisted listening devices "should not be an afterthought" but "considered during the initial rehabilitation/amplification planning stage of treatment".

Rehabilitation programs should also support families and friends or nursing home staff [25,38]. Hearing rehabilitation can either be conducted in a one-to-one setting or within a group, where peer-support and socialisation operate as important rehabilitative tools [17,46].

The benefits of a strong hearing rehabilitation program are numerous. If counselling is offered when a hearing impairment is first diagnosed, it may help with hearing aid retention. This is because poor use of hearing aids is due partly to a lack of understanding of the consequences that hearing loss has for an individual's social, emotional and physical wellbeing [18]. Prior to aid fitting, rehabilitation moderates excessive expectations of hearing aids, mitigating dissatisfaction with the device and lessening the risk of hearing aid rejection [41].

Counselling is more successful if it includes a support person who understands both the reality of the hearing loss and is able to assist with the treatment path being followed [25]. According to Commonwealth Government guidelines, audiologists must provide hearing rehabilitation services as an aspect of their clinical practice [42,47,48]. Despite the Federal government guidelines, audiologists have complained that, under the current funding model, they cannot provide adequate rehabilitation and counselling for each patient [35].

The combination of hearing aid provision and audiologic rehabilitation has been shown to give a better return than the provision of hearing aids alone [17]. However, current hearing rehabilitation programs in Australia are limited and need to be better resourced, organised and accessible to cater for the 1.5 million people in Australia in need of their support [2]. The federal and state governments need to ensure that in depth hearing rehabilitation programs are implemented throughout the Australian community. This could be achieved by the federal government broadening the operation of the Commonwealth Hearing Services Program, or by state governments working through and supporting community-based organisations.

### Screening and education

Screening and case-finding programs allow early and effective intervention. They can be used to identify hearing loss at a younger age and may encourage people who otherwise lack the confidence and self-motivation to have their hearing tested [24]. Currently, the Royal Australian College of General Practitioners (RACGP) recommends that patients over 65 should be offered testing for hearing impairment each year when they attend a general practitioner for other reasons [49]. The recommendations for case-finding produced by the RACGP are guidelines only and compliance and effectiveness has not been assessed, although it is a general rule that case-finding (located in the context of a therapeutic relationship) is more cost-effective than population-based screening. [11,50].

Screening is currently conducted through various government and community-based schemes, such as those offered by Australian Hearing and Better Hearing which travel to various locations around the country and offer free hearing testing [51]. However, these programs are not systematically implemented throughout the Australian population, their success at meeting the needs of the target population is not assured and they have no automatic link to action if the need for action is detected. The current screening and case-finding programs operating in Australia should be evaluated to determine their rates of success and the evidence reviewed as to the likely benefit of greater investment in these activities. In general, the benefits expected from screening for disease and defects have usually not been delivered in practice.

Education about the frequency and impact of hearing impairment amongst older people could be expanded and made one element of a hearing health policy for Australia [18,52]. Education needs to be two-fold, operating at both community and individual levels. At a personal level, education includes a comprehensive pre- and post-fitting rehabilitation program [20]. At a community level, education might involve the kinds of public education campaigns for cervical cancer, which promote both awareness of the significance of the issue and inform the population about screening procedures.

### The cost and effectiveness of audiological rehabilitation

Studies from the United States and the Netherlands have discussed the relation of cost of audiological rehabilitation to the benefits of intervention for hearing impairment [17,53,54]. The value of the effectiveness of hearing aids and hearing rehabilitation programs for treating hearing impairment generally exceeds the human and financial costs associated with the problem. Among an older Dutch population, Joore et al. (2003) determined that the cost of fitting hearing aids was fully justified in terms of benefits gained from the device [53]. Abrams et al. (2002) examined hearing aid use alone, and the use of hearing aids in conjunction with group-based audiological rehabilitation in American subjects [17]. The combination of hearing aid fitting and audiological rehabilitation was more cost-effective than hearing aid use alone [17]. No studies of the economic value of audiological rehabilitation programs have been conducted in Australia. The findings of these overseas studies should be broadly applicable to the Australia but Australian research may reveal nuances and be more persuasive in achieving public funding for these purposes.

## Conclusion

Considering the large numbers of Australians affected by age-related hearing impairment, it is surprising that more has not already been done by both federal and state governments to develop policies. The population who suffer from age-related hearing loss is generally a politically weak one who, possibly because of the nature of their hearing loss may find difficulty in effectively lobbying for policy change.

This literature search has identified several important areas that need to be addressed in the construction of a comprehensive hearing health policy for Australia. These areas are early intervention, hearing aid expense, the use of assisted listening devices, rehabilitation, screening and education. Given the burden imposed by untreated or under treated hearing loss amongst Australians over 55, a strong and coordinated policy response to the issues should be developed for Australia.

Clearly, given the enormity of the problem and the insufficient nature of current treatment options, hearing loss needs to become a priority for Australian Federal and State governments [9]. The Australian Deafness Forum and Better Hearing have suggested that hearing be nominated as an Australian National Health Priority Area (NHPA) [4,31]. The setting of NHPAs by the federal government focuses attention on particular health issues, raising their public profile and increasing the research available to direct government policy. For example, diabetes was positioned as an NHPA in 1996, and this has led to increased research focus on diabetes and national strategies for diabetes management such as through the National Diabetes Strategy [56,27]. More recently in August 2005 dementia was made an NPHA in a move that the government hopes will "boost the national effort involved with the assessment, management, treatment and care of people with dementia" [7]. If age-related hearing impairment were made an NHPA this would emphasize its significance for the 1.5 million Australians who suffer from it and direct government resources and research funding to it.

More research is needed into most aspects of the treatment of age-related hearing impairment in Australia. Current research undertaken by Australian Hearing is important and relevant, but misses large areas identified in this literature search. Gaps in our knowledge of this problem in Australia include the impact of cost of hearing aids and devices on those unable to access free government hearing services, the utility of current hearing screening and audiological rehabilitation programs.

It is 20 years since the World Health Organisation launched its *Prevention of Deafness and Hearing Impairment Program*, with the goal of halving preventable hearing impairment by the year 2010 [55]. This program emphasised the provision of hearing aids to older people but its aims seem to have been largely forgotten in Australia [23]. Current Australian government approaches to age-related hearing impairment need to be built upon through a comprehensive hearing health policy, based upon strong research, to achieve that goal.

## Competing interests

The author(s) declare that they have no competing interests.

## Authors' contributions

JS, PM, JJW and SRL conceived and designed this study. JS conducted the literature search and analysis. JS constructed the draft of the paper. PM, JJW and SRL made significant contributions to the revision of the draft. All authors read and approved the final manuscript.
